# Transformer-based deep learning model for predicting fNIRS short-channel signals

**DOI:** 10.1117/1.NPh.12.4.045008

**Published:** 2025-11-14

**Authors:** Sabino Guglielmini, Vittoria Banchieri, Felix Scholkmann, Martin Wolf

**Affiliations:** a University Hospital Zurich, University of Zurich, Biomedical Optics Research Laboratory, Department of Neonatology, Zurich, Switzerland; b University of Bern, Institute of Complementary and Integrative Medicine, Bern, Switzerland; c University of Zurich and ETH Zurich, Neuroscience Center Zurich, Zurich, Switzerland

**Keywords:** functional near-infrared spectroscopy, short-channel regression, deep learning, transformer encoder, physiological noise, signal denoising, functional near-infrared spectroscopy preprocessing

## Abstract

**Significance:**

Functional near-infrared spectroscopy (fNIRS) enables portable and noninvasive monitoring of cerebral hemodynamics, but hemodynamic changes originating from extracerebral tissues may influence the signals. To avoid this, short-channel regression (SCR) is widely used, yet physical short-separation detectors are not always available or optimally positioned due to hardware limitations or the experimental setup. In such cases, a virtual, data-driven alternative to physical short-channel detectors may be a viable solution.

**Aim:**

We aimed to (i) develop a transformer-based deep learning model to predict short-separation optical density (OD) signals from long-separation channels and (ii) evaluate whether these virtual signals enable effective SCR.

**Approach:**

We trained the model on a resting-state fNIRS dataset (69 subjects) with paired short- and long-separation recordings. Dual-wavelength OD signals in segmented time windows were used as input for a transformer encoder trained to reconstruct the extracerebral hemodynamic component measured by short channels. Model performance was evaluated using 3 independent datasets: a holdout subset of the same resting-state dataset (23 subjects), a second dataset acquired using a different system (40 subjects), and a task-based finger-tapping dataset (4 subjects). A wavelet coherence-based channel rejection step was optionally applied during preprocessing. Predictions were evaluated using signal similarity metrics (mean squared error [MSE], normalized MSE [NMSE], and Pearson correlation [r]) and denoising efficacy (residual variance after regression).

**Results:**

Predicted short-channel signals showed high correspondence with ground-truth measurements in OD (median r=0.70 and NMSE=0.047) and concentration data (up to r=0.67). When used for SCR, virtual regressors effectively denoise long-channel data. Performance was robust across all datasets, with greater accuracy when low-coherence channels were excluded. In motor task blocks, predicted regressors preserved task-evoked activations and reduced residual variance.

**Conclusion:**

Transformer-based models accurately reconstruct extracerebral hemodynamic signals from long-separation fNIRS data, providing a virtual alternative to physical short channels and supporting standardized, hardware-independent preprocessing.

## 1 Introduction

Functional near-infrared spectroscopy (fNIRS) is an optical neuroimaging technique that uses near-infrared light to noninvasively monitor brain hemodynamics.^1^^–^^3^ Because biological tissues are relatively transparent in the 650 to 1000 nm range, light penetrates the scalp and skull and is detected after traversing cerebral tissue.^4^^–^^6^ Over the past few decades, fNIRS has evolved from single-point measurements to high-density imaging arrays and has emerged as a popular neuroimaging modality.^7^^–^^9^ Its portability and relatively low cost make it a safe and practical alternative to functional magnetic resonance imaging (fMRI), enabling studies in naturalistic settings across domains such as cognition, clinical neuroscience, and brain-computer interfaces.^10^^–^^12^

A methodological challenge in fNIRS is its sensitivity to hemodynamic changes in extracerebral tissue (including the scalp and skull), which is highly vascularized.^13^ These tissue layers add physiological signals, including cardiac pulsations, respiratory cycles, and low-frequency blood pressure oscillations (e.g., Mayer waves) to the cortical signals. If these confounding signals are not properly handled, they reduce the signal-to-noise ratio (SNR) and lead to misinterpretation of the fNIRS data such as false negative and false positive interpretations, i.e., falsely not detecting brain activity, which is masked by physiological noise or falsely interpreting physiological noise as brain activity.^13^^–^^15^

To address this, researchers developed the short source–detector separation channel approach, widely known as short-channel regression (SCR). In SCR, additional optodes are placed at a short source–detector separation (typically 8 to 15 mm in adults) to record signals that are almost exclusively from the superficial layer.^16^ These short-separation channels (also called short-distance or short-separation measurements) have negligible sensitivity to cortical hemodynamics but capture the systemic fluctuations in scalp blood concentration. Saager and Berger first proposed the concept using two-layer Monte Carlo simulations showing that measuring the optical signal at a short separation (a few millimeters, sampling only the scalp) and performing a least-squares fit of this shallow signal to the co-localized long-separation signal, effectively subtracts the scalp component and recovers the bottom-layer brain signal.^17^ Gagnon et al. systematically varied the distance between short- and long-separation optodes and demonstrated that superficial interference is spatially inhomogeneous across the scalp. Their findings indicate that a short-separation channel provides the greatest benefit to the spatially adjacent long-separation channel.^18^ As a result, the improvement from SCR deteriorates as the distance between the short-separation optode and the target channel increases. These insights motivated high-density probe designs pairing each long channel with a proximal short channel. Consistent with this, Monte Carlo simulations by Brigadoi and Cooper determined that the optimal source-detector spacing for short channels in adults is ∼8.4  mm (yielding ∼5% relative sensitivity to brain tissue).^16^ Yücel et al. reported that applying the SCR approach not only increases the statistical significance of detected activations but also yields better-localized cortical responses across a range of tasks with differing autonomic responses.^19^ As we demonstrated in previous work, the SCR approach improves the reproducibility of fNIRS measurements and results and is especially powerful when heterogeneous scalp hemodynamics are considered.^20^^,^^21^ The SCR approach has become the best practice in the field.^22^ When SCR is performed in combination with the systemic physiology augmented fNIRS (SPA-fNIRS) methodology, which was developed by our groups in Bern and Zurich, fNIRS measurements are obtained with increased physiological interpretability and a greater chance of detecting neurovascular-coupling–based changes.^23^

The advent of SCR has enabled more reliable fNIRS recordings across various research domains. In brain–computer interface (BCI) studies, where fNIRS is used to classify mental states or commands, removing systemic noise is vital for improving classification accuracy. Many fNIRS-BCI pipelines now include short-separation channels to preprocess the signals in real time, enabling true (i.e., cerebral) hemodynamic patterns to be fed into machine learning (ML) classifiers.^24^ In neurofeedback contexts, where participants self-regulate brain activity with fNIRS signals as feedback, SCR ensures that the feedback reflects cortical changes rather than global arousal fluctuations. Preliminary fNIRS neurofeedback studies have begun to adopt short channels or related filtering to improve the specificity of the neurofeedback signal. This is expected to enhance training efficacy and reliability.^25^ In addition, resting-state functional connectivity studies with fNIRS have greatly benefited from SCR. Emerging research has demonstrated that global superficial physiological signals distort functional connectivity estimates in fNIRS by introducing widespread, non-neuronal correlations across the scalp. These spurious fluctuations not only inflate functional connectivity values but also compromise reliability across sessions.^26^ However, applying correction techniques such as SCR greatly reduces these artifacts. Consequently, the underlying neuronal connectivity is more accurately represented, with fNIRS-derived resting-state networks showing strong correspondence to those measured with fMRI in the same participants.^27^

Despite its clear advantages, SCR is not a perfect solution and has important limitations. One concern is that, while short channels mostly sample scalp vasculature, they may still capture a small amount of cortical signal. Consequently, regressing it out could slightly attenuate the true neural signal, introducing a modest bias. A second issue is the assumption that the short channels are representative of the superficial signal in the long channel. In practice, however, scalp hemodynamics is heterogeneous and varies across different scalp regions. If a short channel is too far from a long channel, or if one short channel is assumed to serve an entire region, it may not perfectly capture the local scalp noise affecting the long channel.^18^ Another practical issue is the noise and SNR of short channels. Short-separation measurements have much stronger detected light intensity (due to the short pathlength), but often have a smaller physiological signal amplitude (because they sample a tiny tissue volume). They also suffer from detector saturation or light leakage at very small separations. Thus, the short-channel signals themselves may have a low SNR, and improper handling (or overfitting when regressing multiple noisy short channels) could reintroduce noise. Third, both short-separation and long-separation channels are similarly influenced by systemic physiological factors such as blood pressure or changes in arterial ${\mathrm{CO}}_{2}$ concentration (${\mathrm{PaCO}}_{2}$).^28^ Because these fluctuations affect cerebral and extracerebral vasculature in parallel, the short-channel signal does not provide a differential measure of superficial noise. As a result, SCR alone cannot successfully remove this type of systemic interference, and additional approaches such as SPA-fNIRS are required.

In the absence of short channels or to enhance noise correction, several alternative signal processing methods have been applied in fNIRS. Statistical decomposition methods, such as principal component analysis (PCA) and independent component analysis, aim to isolate and remove dominant noise components. However, their effectiveness depends on assumptions about the independence or orthogonality of neuraly induced and systemic physiological signals.^29^^–^^31^ Auxiliary physiological signals are also used as regressors in general linear models to account for systemic influences.^25^^,^^26^^,^^32^ Building on this, the temporally embedded canonical correlation analysis framework identifies components shared between fNIRS and auxiliary signals, yielding more accurate denoising.^33^^,^^34^ This method outperforms standard SCR in multiple benchmarks, including signal fidelity and detection sensitivity. Additional strategies include adaptive filtering, Kalman filtering for real-time correction, wavelet-based motion artifact removal, and combined spatial-frequency domain filtering.^25^^,^^35^^,^^36^ No single method is universally optimal, and hybrid approaches often yield the best results. For instance, combining band-pass filtering, PCA, and SCR has proven highly effective. A comparative study by Santosa et al. found that multi-regressor short-channel correction was the most effective method, whereas PCA on baseline data was the best alternative when short channels were unavailable.^25^

As data-driven techniques have become more prevalent, researchers have started to use ML and deep learning to enhance fNIRS signal cleaning and brain signal extraction. One approach involves using supervised learning to explicitly model the mapping between noisy and clean signals.^37^^,^^38^ For instance, Zhi et al. recently introduced a generative deep learning model to reconstruct damaged fNIRS signals, such as those corrupted by motion or with a low SNR.^39^ Other studies have used deep learning for automated artifact detection and removal. For instance, convolutional neural networks (CNNs) have been trained to identify segments of fNIRS data containing motion artifacts for exclusion or correction.^40^^,^^41^

In recent years, transformer-based architectures, which were originally popularized in natural language processing, have begun to gain traction in the analysis of physiological time series.^42^^–^^44^ Transformers utilize self-attention mechanisms that allow the model to dynamically weigh and integrate information from all time points. This makes them particularly effective at capturing long-range temporal dependencies and subtle signal patterns. This contrasts with traditional recurrent neural networks, which rely on sequential processing and often struggle with long-term dependencies.^45^ Such capabilities have led to successful applications in EEG, where transformer encoders improve performance in tasks such as emotion detection and motor imagery classification.^46^^,^^47^ These architectural strengths, modeling long-range dependencies, enabling parallel computation and flexibility to include spatial/feature information, make transformer encoders well-suited for estimating short-channel fNIRS signals from multichannel optical density (OD) data. This is particularly relevant since short-channel signals reflect multi-scale systemic oscillations, from cardiac and respiratory rhythms to slower Mayer waves, which require models capable of capturing both short- and long-term dependencies.

In this context, we propose a transformer-based deep learning framework to generate high-fidelity virtual short-channel signals from long-separation fNIRS data. The aim is to enable robust SCR even in the absence of physical short-channel detectors, thereby improving noise removal and preserving the quality of the cortical signal across diverse recording conditions. Specifically, our objectives are to (1) design a transformer encoder tailored for fNIRS time series, (2) train it using resting-state recordings with both long-separation and short-channel data as ground-truth, (3) assess its generalization to unseen resting and task-based datasets, and (4) evaluate its functional impact by comparing denoising performance with that of real short-channel regressors.

## 2 Materials and Methods

### 2.1 Datasets

Data used in this study were drawn from three separate datasets, each of which was collected with distinct experimental setups and protocols. Informed consent was obtained from all participants prior to data collection, and the study protocols were approved by the Ethics Committee of the County of Zurich.

**Fig. 1:**
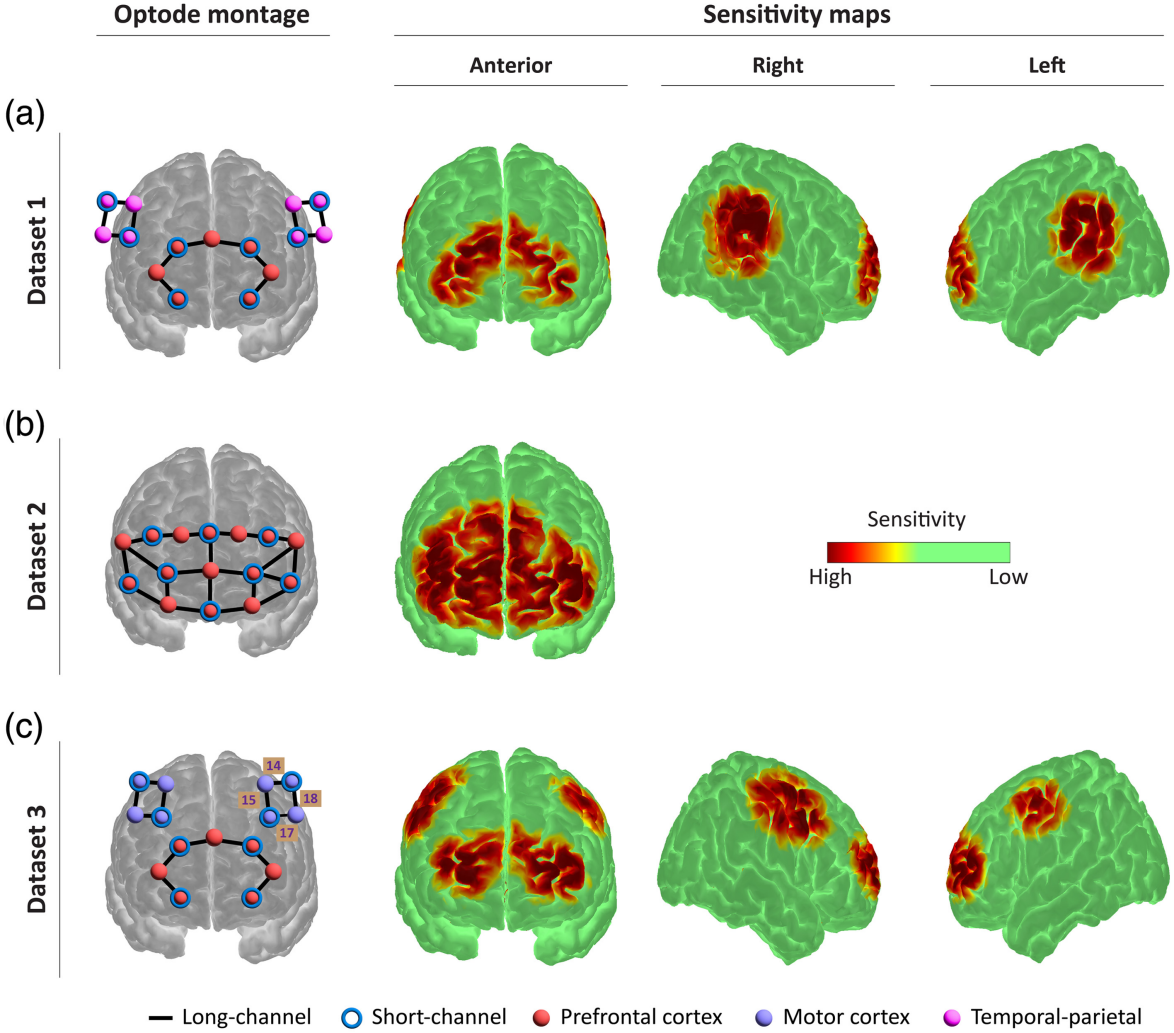
Optode montage layouts and sensitivity maps for the three datasets used in this study. (a) Montage for dataset 1, acquired with the NIRSport 2 system. The array includes 14 long-separation channels covering prefrontal and temporal-parietal areas, each paired with short-separation detectors placed at 8 mm from the source, yielding 8 short-channels in total. On the right, the sensitivity maps show anterior, right, and left cortical coverage. (b) Montage for dataset 2, recorded using the NIRSport 1 system. It comprises 20 long-separation channels and 8 short-separation channels, all positioned over the frontal cortex. On the right, the sensitivity map displays the anterior cortical sensitivity distribution. (c) Montage for dataset 3 including 14 long-separation channels and 8 short-separation channels covering prefrontal and motor cortex areas. The numbered channels indicate the motor cortex regions that were specifically analysed. On the right, the sensitivity maps show anterior, right, and left cortical coverage.

Dataset 1 comprises 92 resting-state fNIRS recordings acquired during a previous hyperscanning study involving healthy adult subjects. The optode montage consisted of 14 long-separation (30 mm) channels, which were distributed as follows: six channels over the prefrontal cortex, four channels over the left temporo-parietal cortex, and four channels over the right temporo-parietal cortex. In addition, eight short-separation (8 mm) channels were implemented, with each short-channel detector bundled adjacent to individual sources (see Fig. 1(a), which shows the optode montage used for dataset 1 alongside the corresponding sensitivity maps for anterior, right, and left cortical regions). During each recording, subjects were instructed to remain seated comfortably, keep their eyes closed, and avoid movement for a continuous 6-min baseline period. Recordings were acquired using the NIRSport 2 system (NIRx Medical Technologies GmbH, Berlin, Germany), which is equipped with eight dual-tip LED sources and eight avalanche photodiode (APD) detectors. Data acquisition was performed with Aurora software (NIRx Medizintechnik GmbH) at two near-infrared wavelengths [760 nm ($\lambda_{1}$) and 850 nm ($\lambda_{2}$)] with a sampling frequency of 8.719 Hz.

Dataset 2 included 40 resting-state fNIRS measurements collected from healthy adult subjects using a NIRSport 1 system (NIRx Medical Technologies GmbH). The montage was composed of 20 long-separation (30 mm) channels, along with eight frontal short-separation (8 mm) channels [see Fig. 1(b), which presents the optode montage used for dataset 2 along with the anterior sensitivity map representing cortical coverage]. Recordings were performed in a resting-state condition lasting 6 min, during which subjects were instructed to minimize movement and maintain a relaxed posture. The NIRSport 1 system utilized eight single-tip LED sources and eight silicon photodiode (SiPD) detectors, sampling at a frequency of 7.812 Hz and at wavelengths of 760 and 850 nm.

Dataset 3 consisted of recordings of four healthy subjects performing a finger-tapping task. The experimental protocol involved a 10-min session, during which the subjects alternated between 20-s periods of tapping their right hand and 20-s periods of rest. The instrumentation was identical to that used for dataset 2: the NIRSport 1 system with eight single-tip LED sources and eight SiPD detectors. The montage configuration, including the associated sensitivity maps for the anterior, right, and left brain regions, is shown in Fig. 1(c).

### 2.2 Preprocessing

**Fig. 2:**
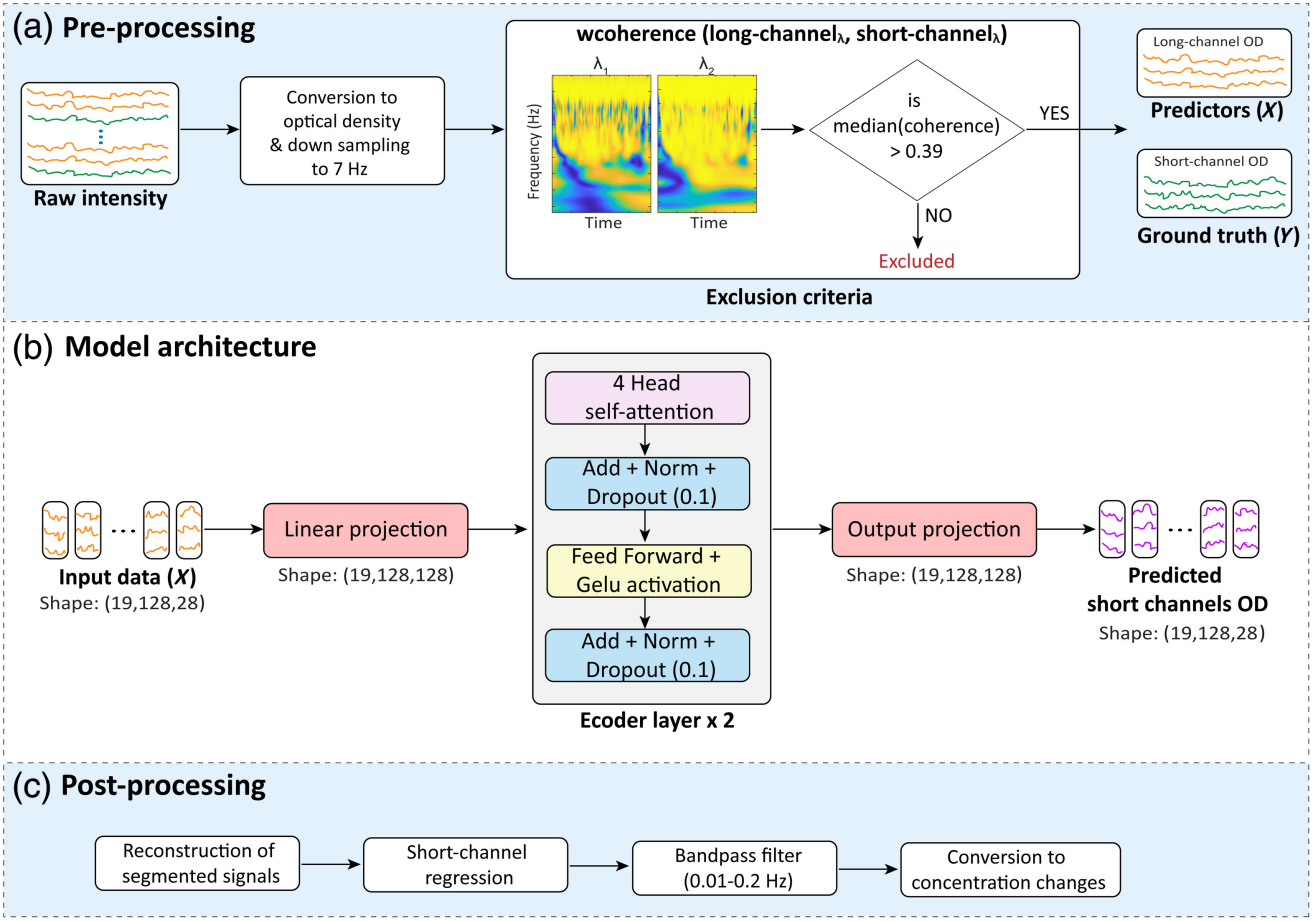
Overview of data preprocessing, model architecture, and post-processing pipeline. (a) Raw fNIRS light intensity data are converted to optical density (OD) and downsampled to 7 Hz. Channels are filtered based on signal quality, using a wavelet coherence threshold to exclude unreliable long–short-channel pairs. The remaining data are split into predictors (long channels) and ground truth targets (short-channels). (b) Model architecture: each 6-min recording is segmented into 19 non-overlapping windows of 128 samples. Each window has dimensions [128×28] (128 time points × 28 features from 14 long-separation channels × 2 wavelengths). For model training, all windows were concatenated across subjects, and the transformer received inputs in the standard format [batch_size, 128, 28], with batch_size = 128. The core of the model consists of two transformer encoder blocks with multi-head self-attention and feed-forward layers. The final output projection generates the predicted short-channel OD time series. (c) Post-processing: predicted segments are reconstructed into a continuous time series. Short-channel regression (SCR) is applied using both predicted and measured short channels. Processed signals are bandpass filtered and converted to concentration changes using the modified Beer-Lambert law.

Figure 2(a) illustrates the preprocessing pipeline applied to the raw fNIRS data. Initially, the raw light intensity signals were converted into OD using the standard Homer2 software function, *hmrIntensity2OD.* Although we tested wavelet-based motion artifact correction on the OD data from long channels using the *hmrMotionCorrectWavelet* function from Homer2 (with a coherence threshold of 0.1),^48^^,^^49^ this procedure did not yield any improvement in the model’s predictive performance. Therefore, to prioritize generalizability and robustness under real-world conditions, we chose not to include this step in the final preprocessing pipeline. Results obtained with motion artifact correction are presented in Tables S1–S3 in the Supplementary Material. To ensure compatibility and consistency across different datasets, the data were uniformly downsampled to 7 Hz. Subsequently, data quality control was applied by filtering out low-quality long–short channel pairs using wavelet coherence analysis.^48^ Wavelet coherence, calculated via the MATLAB *wcoherence* function, provides a robust measure of the similarity between each long channel and its corresponding nearest short channel in both the time and frequency domains. To determine an appropriate median coherence threshold, we evaluated the model performance across several values (0.20, 0.30, 0.39, 0.40, and 0.50), retraining and testing at each level. Based on its optimal balance of signal quality, predictive performance, and data retention, a threshold of 0.39 was selected (see Table S4 in the Supplementary Material). This coherence-based filtering strategy ensures the exclusion of misleading channel pairs (e.g., poor-quality long channels paired with good-quality short channels or vice versa), which would otherwise degrade model training by introducing ambiguous or inconsistent input–output relationships. After quality control, the short-channel OD signals (which serve as the ground truth and are denoted as Y) and the corresponding long-channel OD signals (which are the predictors and are denoted as X) were structured separately into training matrices.

### 2.3 Model Architecture and Training

The proposed model is a transformer encoder architecture that has been adapted to suit the structure of fNIRS time-series data. It was designed to reconstruct short-channel OD signals based on input from corresponding long-channel measurements, as illustrated in Fig. 2(b).

For training purposes, 69 resting-state recordings were used from dataset 1. Each 6-min fNIRS baseline recording (≈2520 samples at 7 Hz) was segmented into 19 non-overlapping windows of 128 samples each (≈18.2  s). The remaining samples at the end of the recording were discarded to ensure that all windows had an identical length. This segment length was selected via a grid search comparing performance across various configurations (see Table S5 in the Supplementary Material), revealing that 128 samples strike the optimal balance between model accuracy, temporal resolution, and computational efficiency. Shorter windows resulted in underfitting due to insufficient temporal context, whereas longer windows increased computational cost without significantly improving performance. Across all 69 subjects, this procedure yielded a total of 1311 windows. Each input sequence consists of multichannel OD signals from 14 long-separation channels, recorded at two wavelengths ($\lambda_{1}$ and $\lambda_{2}$), resulting in 28 features per time step. For each window, input matrices are $X\in R^{19\times 128\times 28}$, where 19 corresponds to the number of windows per subject, 128 to the temporal dimension, and 28 to the feature dimension. The corresponding ground-truth short-channel matrices were denoted as Y. Predictions were generated in the OD domain rather than directly in concentration units, as OD represents the raw measurement domain, avoids dependence on Beer–Lambert conversion parameters, preserves dual-wavelength information, and ensures greater generalizability across acquisition systems. First, the raw OD signals were linearly projected into a 128-dimensional embedding space using a learned affine transformation.^49^ This projection enables the model to map the raw input data into a latent feature space, facilitating the capture of temporal and spatial relationships. This serves as both a dimensionality expansion and a format conversion, making the input suitable for transformer-based encoding. Each projected sequence is then passed through two identical transformer encoder blocks.

Each block of the model comprises two main sublayers. The first is a multi-head self-attention layer with 4 attention heads, followed by a feed-forward neural network with 256 hidden units (corresponding to a 2× expansion over the 128-dimensional embedding) and a Gaussian error linear unit (GELU) activation function. This approach aligns with the original transformer design principle of increasing the capacity by a factor of 2 to 4 to ensure sufficient nonlinearity for temporal and feature-level mixing.^50^

To ensure stable and effective training, both sublayers are enclosed within a residual learning framework comprising three key components: (i) a residual connection (add), (ii) layer normalization (norm), and (iii) dropout regularization. After each sublayer, the model applies a residual connection that adds the original input of the sublayer to its output. This preserves the input signal and facilitates the learning of incremental refinements rather than overwriting information entirely. These residual pathways are known to ease optimization and prevent vanishing gradients, particularly in deep architectures.^51^^,^^52^ Following the residual summation, layer normalization is applied. The norm step standardizes the summed signal across features to stabilize activation dynamics, leading to faster convergence and more consistent gradient flow.^53^^,^^54^ Centering and scaling the inputs to each layer makes the model more resilient to shifts in distribution during training.^55^ Finally, dropout with a probability of 0.1 is used as a form of regularization.^56^ By randomly deactivating 10% of the hidden units during each forward pass, the model is discouraged from relying too heavily on any single unit. This promotes generalization and reduces overfitting, which is particularly important when learning from physiological signals with inherent variability.^57^^,^^58^ Together the add, the norm, and the dropout structures are repeated after both the self-attention and feed-forward subcomponents in each encoder block. This ensures that the learned representations remain stable, interpretable, and robust throughout the network.

After encoding, the output is passed through a linear decoder layer to map it back to the original signal space (i.e., the 28-dimensional OD vector corresponding to the predicted short-channel signals). This output projection translates the latent feature space into interpretable physiological signals. Thus, the model learns a direct mapping from long-channel OD time series to the corresponding short-channel OD traces, functioning as a virtual short-channel estimator. The predicted outputs are then concatenated across all windows and used either for direct analysis or as inputs in SCR pipelines.

The model was trained on dataset 1 with a batch size of 128 for 180 epochs. The 1311 windows were randomly partitioned into training (80%, 1048 windows), validation (10%, 131 windows), and test (10%, 132 windows) subsets. Training was performed using PyTorch v2.1 on an NVIDIA T4 GPU provided by the Google Colab environment. Model parameters were optimized using the AdamW optimizer with an initial learning rate of 0.0008.^43^ A cosine annealing scheduler dynamically adjusted the learning rate throughout training to ensure effective convergence.

To achieve accurate short-channel predictions in terms of both magnitude and temporal profile, a hybrid loss function was employed, combining mean squared error (MSE) and Pearson correlation (r):

Ltotal=MSE(y^,y)+α*(1−r(y^,y)),

where y^ is the predicted short-channel OD signal, y is the ground-truth short-channel OD signal, MSE(y^,y)=1n∑i=1n(yi^−yi)2 quantifies the average prediction error over n time points and channels, r(y^,y) denotes the Pearson correlation coefficient capturing temporal similarity, and α=0.3 is a weighting factor that balances magnitude error minimization with temporal shape preservation.

To further enhance the robustness and generalization performance of our transformer model during inference, we employed test-time augmentation. For each input window, we generated five slightly perturbed versions by introducing small variations including Gaussian noise injection (σ=0.01), temporal jittering (±10 samples), amplitude scaling (±5%), and baseline shifts.^59^^–^^62^ These perturbations were designed to mimic moderate, physiologically plausible variability (e.g., baseline drift, minor motion, sensor fluctuations) rather than severely corrupted signals. Predictions were computed independently for each augmented input, and the final short-channel signal was obtained by averaging these outputs. This ensemble-based approach effectively reduces prediction variance and yields smoother estimates of short-channel signals, particularly in conditions involving elevated noise levels or minor temporal misalignments, which are commonly encountered in fNIRS measurements.

### 2.4 Testing Datasets and Model Evaluation

The trained model was evaluated on three separate datasets to assess its predictive performance and ability to generalize across different experimental conditions and setups.

To evaluate the initial performance of the model, we utilized a subset of dataset 1 consisting of 23 resting-state recordings. The signal preprocessing pipeline was identical to that described for the training dataset (see Sec. 2.2), resulting in testing matrices with dimensions 19×128×28.

Dataset 2 differed from the configuration of the training dataset. Two distinct analyses were conducted: (i) preprocessing identical to the training pipeline, including wavelet coherence-based channel rejection, and (ii) preprocessing without channel rejection. This dual approach enabled us to evaluate the robustness of the model on both carefully filtered and potentially noisy data. The testing matrices generated had dimensions of 11×128×40 and presented two primary challenges: (i) compatibility with a different acquisition system and (ii) managing an increased number of channels compared with the training setup. Transformer architectures inherently require a fixed number of input channels that match their training configuration, which makes direct deployment to datasets with a different number of channels challenging. Practical solutions include retraining the model entirely or fine-tuning an existing model. Here, we adopted the latter, a computationally efficient strategy involving: (i) extending input and output layers to match the new channels count, (ii) retaining the pre-trained weights for the channels that match the original configuration, (iii) randomly initializing the new weights that correspond to additional channels, and (iv) conducting brief fine-tuning on a subset of dataset 2 (20 recordings), minimally altering the core transformer weights to retain the previously learned features. This procedure adapted the model efficiently to manage the expanded 40-channel configuration while preserving the acquired knowledge.

To further evaluate model generalization, we analyzed data from a task-based condition involving finger tapping (dataset 3). Four participants performed 20-s intervals of rest and right-hand finger tapping, alternating every 20 s, for 10 min, using the NIRSport 1 system. Preprocessing replicated the pipeline applied to dataset 2, generating predictor matrices of dimensions 33×128×28. We examined the block-averaged hemodynamic responses elicited during the finger-tapping task across four predefined left motor cortex channels (channels 14, 15, 17, and 18) in each of the four subjects.

### 2.5 Predictions Post-processing

The model-predicted short-channel OD signals were initially reconstructed into continuous signals by merging the windows. A SCR was then performed using the *hmrSSR* function twice for each dataset: first with the actual short-channel signals, and then with the model-predicted signals, to enable direct comparison.

After the SCR, identical processing was applied to both the actual and predicted short-channel data using Homer 2 functions. The long-channel signals were bandpass filtered between 0.01 and 0.2 Hz using the *hmrBandpassFilt* function.^63^ The filtered OD signals were then converted into concentration changes using the *hmrOD2Conc* function with a differential path length factor (DPF) set to 6. The post-processing steps are depicted in Fig. 2(c).

To quantify task-related hemodynamic responses, the block-averaged signal was calculated from the processed concentration data of dataset 3 ($\left[O_{2}\mathrm{Hb}\right]$ and [HHb]). The signals were segmented into predefined task and rest intervals using the Homer2 function *hmrBlockAvg*. The resulting averaged $\left[O_{2}\mathrm{Hb}\right]$ and [HHb] values were generated for subsequent interpretation.

### 2.6 Performance Metrics

To quantitatively assess the performance of the model in predicting short-channel signals and their utility in noise regression, we employed four complementary metrics:

• **Mean squared error (MSE)** was calculated as the average of the squared differences between the predicted and the true OD signals across all channels and time points. This metric reflects pointwise accuracy, where lower values indicate that the predicted signal closely follows the ground-truth in absolute magnitude.
• **The Pearson correlation coefficient (*****r*****)** was computed for each pair of predicted and ground-truth OD signals to evaluate how well the model preserved the shape and temporal dynamics of the signals. Values closer to 1 indicate a stronger linear correspondence in the overall time course, regardless of scale.
• **Normalized mean squared error (NMSE)** was derived by dividing the MSE by the square of the dynamic range of the ground-truth signal (i.e., the difference between its maximum and minimum value). This normalization ensures comparability across signals with different amplitude scales and subject-specific variability. A low NMSE thus indicates that the prediction error is small relative to the natural range of fluctuations in the true signal.
• **Residual variance** was calculated on long-separation channels after SCR using either the predicted or the ground-truth short-channel signals. For each channel and chromophore, we computed the variance of the denoised time series. Lower residual variance indicates more effective removal of superficial physiological noise, and hence a more successful regression process.

All metrics were computed per subject and channel and then aggregated by taking the median across channels and subjects to produce group-level performance summaries. We evaluate the performance of the model in each dataset. The analysis focused on three key objectives: (i) assessing the quality of the prediction of short-channel OD signals, (ii) evaluating the accuracy of hemoglobin concentration estimates derived from the predictions of short-channel signals, and/or (iii) testing the efficacy of the denoising of the prediction of short-channels signals when used in SCR.

## 3 Results

### 3.1 Dataset 1

First, we evaluated the performance of the model on a held-out subset of 23 resting-state recordings from dataset 1. The aim of this evaluation was to determine the extent to which the model predicts short-channel signals from long-channel inputs, both in terms of OD and derived chromophore concentrations. In addition, we assessed the effectiveness of using these predicted short-channel signals as substitutes for ground-truth short-channels in SCR for denoising cortical measurements.

Table 1 reports the prediction performance in the OD domain. The predicted short-channel signals closely resembled the ground-truth signals, achieving a median Pearson correlation coefficient of 0.7019 and an NMSE of 0.0471.

**Table 1:** Performance of predicted versus actual short-channel OD signals. Metrics are median values of mean squared error (MSE), normalized MSE (NMSE), and Pearson correlation coefficient (r) across subjects.

Metric	MSE	NMSE	r
OD	0.0001	0.0471	0.7019

To assess whether the predicted short-channel signals preserved physiologically meaningful trends, we converted the OD data into chromophore concentrations using the modified Beer–Lambert law. As shown in Table 2, the predicted signals remained consistent with the ground-truth concentrations. The best correspondence was observed for [tHb] (r=0.6664), followed by $\left[O_{2}\mathrm{Hb}\right]$ and [HHb] (r=0.6216 and r=0.4891, respectively).

**Table 2:** Performance of predicted versus actual short-channel concentrations ($\left[O_{2}\mathrm{Hb}\right]$, [HHb], [tHb]) after OD-to-concentration conversion. Metrics quantify signal fidelity for each chromophore.

Type	MSE	NMSE	r
$\left[O_{2}\mathrm{Hb}\right]$	$5.53\times 10^{-14}$	0.0314	0.6216
[HHb]	$1.38\times 10^{-14}$	0.0288	0.4891
[tHb]	$9.31\times 10^{-14}$	0.0263	0.6664

Next, we evaluated the functional utility of the predicted short-channel signals as regressors for SCR. Table 3 shows that when used in SCR, the predicted regressors were as effective as the short-channel ground-truth at suppressing superficial physiological noise from long-channel signals. For example, the cleaned long-channel signals showed strong correlations with the signals regressed using ground truth, particularly for [tHb] and [HHb], with values exceeding r=0.76. Although tHb is often expected to resemble $O_{2}\mathrm{Hb}$, in our results, its correlation patterns aligned more closely with HHb, likely reflecting that the reported metrics capture variance and temporal dynamics beyond amplitude alone.

**Table 3:** Performance comparison of long-channel signals following short-channel regression (SCR), using either predicted or ground-truth short-channel regressors. Metrics reflect how well the model supports signal cleaning.

Type	MSE	NMSE	r
$\left[O_{2}\mathrm{Hb}\right]$	$1.77\times 10^{-14}$	0.0140	0.7267
[HHb]	$5.09\times 10^{-15}$	0.0134	0.7634
[tHb]	$9.79\times 10^{-15}$	0.0113	0.7684

To further quantify the effectiveness of SCR, we computed the mean residual variance in long-channel signals post-regression. As summarized in Table 4, regression using predicted short-channel signals resulted in lower or comparable residual variance across all chromophores.

**Table 4:** Mean residual variance (Var) in long-channel signals post-SCR using ground-truth vs. predicted short-channel regressors. Lower values indicate better noise suppression.

Type	Ground-truth Var	Predicted Var
$\left[O_{2}\mathrm{Hb}\right]$	$3.01\times 10^{-14}$	$2.74\times 10^{-14}$
[HHb]	$1.07\times 10^{-14}$	$1.03\times 10^{-14}$
[tHb]	$1.27\times 10^{-14}$	$1.08\times 10^{-14}$

**Fig. 3:**
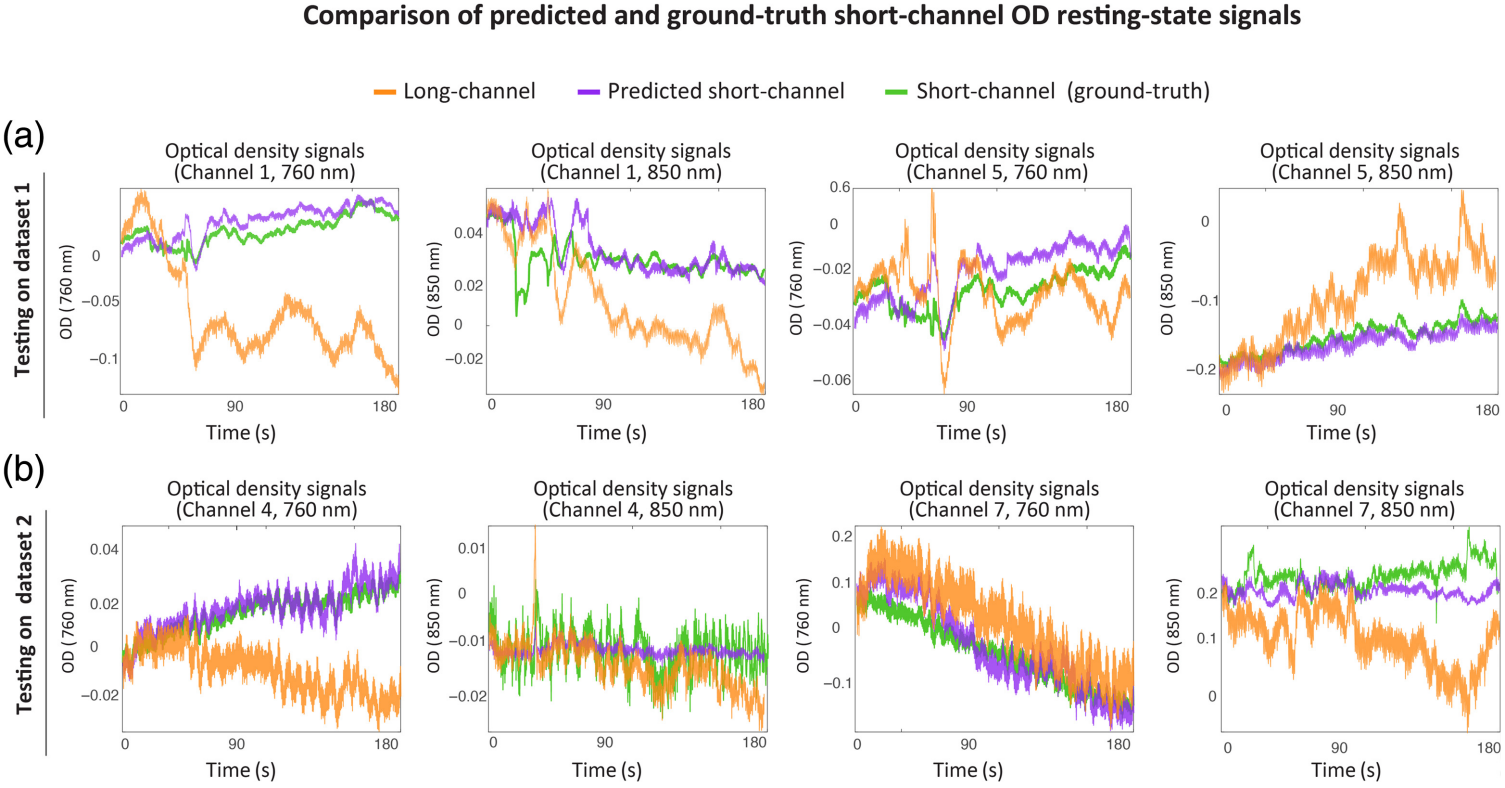
Example optical density (OD) time series from two representative resting-state datasets. (a) Traces from dataset 1 show the long-channel OD (orange), ground-truth short-channel OD (green), and predicted short-channel OD (purple) for a representative segment. (b) Traces from dataset 2, acquired using a different fNIRS system and optode layout, show comparable alignment between predicted and measured short-channel OD. These plots demonstrate that the model captures superficial hemodynamic trends with high fidelity, even across systems and preprocessing conditions.

**Fig. 4:**
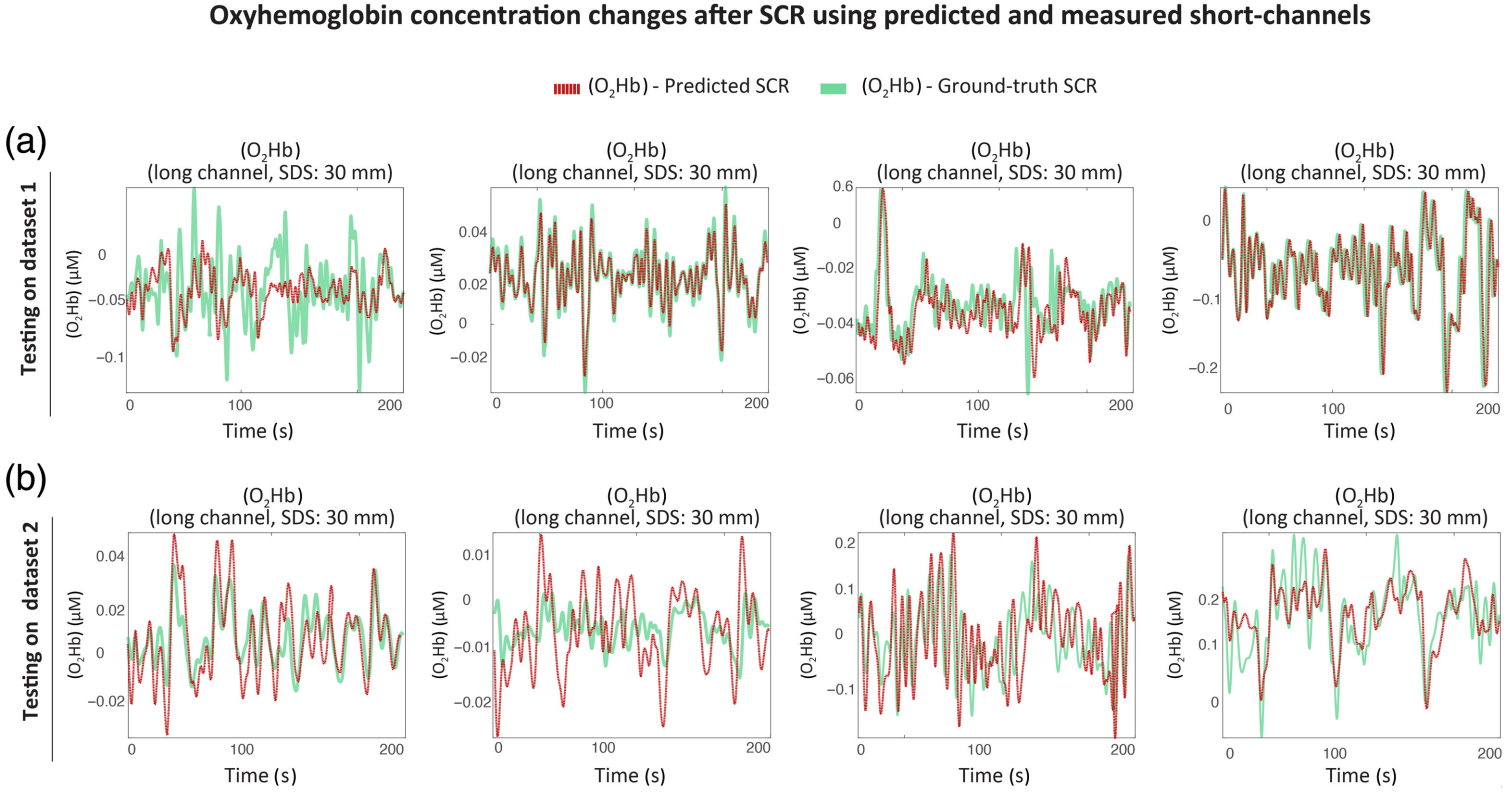
Time courses of oxygenated hemoglobin concentrations ($\left[O_{2}\mathrm{Hb}\right]$) following short-channel regression (SCR) using either predicted or ground-truth short-channel data. (a) Results from dataset 1 illustrate that predicted short-channel signals yield $\left[O_{2}\mathrm{Hb}\right]$ dynamics nearly indistinguishable from those obtained using real short-channel regressors. (b) Results from dataset 2 show consistent behavior despite differences in hardware and optode layout.

Qualitative inspection of signal traces also confirms the fidelity of the predicted short-channel outputs. Figure 3(a) shows representative OD traces from dataset 1, with the predicted signals closely aligning with both the long-channel signals and the ground-truth short-channels. The long-channel signal (orange), the ground-truth short-channel (green), and the predicted short-channel signals (purple) are plotted together, clearly showing the overlap between the predicted and actual short-channel signals. Figure 4(a) shows the impact of SCR on $\left[O_{2}\mathrm{Hb}\right]$ concentrations. The close alignment between the red (predicted-based SCR) and green (real short-channel-based SCR) traces demonstrates that virtual short channels effectively clean task-unrelated fluctuations.

### 3.2 Dataset 2

To assess the generalizability of the transformer model across different acquisition systems and optode configurations, we tested it on dataset 2. As shown in Table 5, preprocessing was critical for signal fidelity. Without rejecting poor-quality channels, the model achieved an NMSE of 0.1784 and a Pearson correlation coefficient of only 0.2771. After applying channel rejection, the NMSE decreased by over 50% to 0.0875, and the correlation improved to 0.4296.

**Table 5:** OD prediction metrics with and without wavelet coherence-based channel rejection.

Preprocessing	MSE	NMSE	r
Without rejection	0.00173	0.1784	0.2771
With rejection	0.00046	0.0875	0.4296

Next, we evaluated prediction performance in the hemoglobin concentration domain, again under both preprocessing conditions. Table 6 summarizes these results. When no channel rejection was applied, correlation values were modest, particularly for [HHb] (r=0.3095) and [tHb] (r=0.4160). However, with channel rejection, all three chromophores showed substantial improvement. Notably, the correlation for [tHb] increased to 0.6099.

**Table 6:** Short-channel concentration prediction performance without channel rejection. Results show how noise impacts chromophore prediction.

Type	Without rejection			With rejection		
	MSE	NMSE	r	MSE	NMSE	r
$\left[O_{2}\mathrm{Hb}\right]$	$1.14\times 10^{-12}$	0.0541	0.3640	$5.5\times 10^{-13}$	0.0342	0.4999
[HHb]	$1.26\times 10^{-12}$	0.1768	0.3095	$4.7\times 10^{-13}$	0.0497	0.5083
[tHb]	$8.74\times 10^{-13}$	0.0372	0.4160	$4.8\times 10^{-13}$	0.0188	0.6099

A visual inspection of the example traces shown in Fig. 3(b) provides further support for these metrics. It shows that the predicted short-channel OD signals align well with ground-truth signals, especially following rejection of low-coherence channels. The long-channel OD signals are plotted in orange, the ground-truth short-channels in green, and predicted short-channel signals in purple. Similarly, Fig. 4(b) presents denoised $\left[O_{2}\mathrm{Hb}\right]$ concentration time courses after SCR, highlighting the ability of predicted regressors to support effective physiological noise removal. The $\left[O_{2}\mathrm{Hb}\right]$ values obtained using the predicted signal (dashed red) with the SCR closely match those obtained using the ground-truth regressors (green), demonstrating the utility of the model in downstream denoising even under domain shift.

### 3.3 Dataset 3

Dataset 3 assessed the performance of the model during a motor task (finger tapping), focusing on short-channel prediction, chromophore concentration estimation, and denoising efficacy in activation-relevant long channels.

First, we analyzed the raw OD signal predictions, both with and without wavelet coherence-based channel rejection. As shown in Table 7, applying channel rejection substantially improved prediction accuracy. Without rejection, the predicted short channels showed weak correspondence with the ground truth (Pearson r=0.0764, NMSE=0.1651). Once preprocessing excluded unreliable channels, performance improved markedly (r=0.4061, NMSE=0.0408).

**Table 7:** OD prediction accuracy during task execution with and without channel rejection.

Preprocessing	MSE	NMSE	r
With rejection	0.03142	0.0408	0.4061
Without rejection	0.00153	0.1651	0.0764

Next, we evaluated the accuracy with which the OD signals could be converted into changes in hemoglobin concentration in short channels, specifically those related to motor cortex activation. As shown in Table 8, the predicted $\left[O_{2}\mathrm{Hb}\right]$ and [tHb] values exhibited moderate agreement with respective correlation values of 0.4809 and 0.3100. However, [HHb] exhibited poor correspondence (r=−0.1021).

**Table 8:** Short-channel concentration prediction metrics for activation-relevant channels ($\left[O_{2}\mathrm{Hb}\right]$, [HHb], [tHb]). These results assess signal fidelity during motor tasks.

Type	MSE	NMSE	r
$\left[O_{2}\mathrm{Hb}\right]$	$8.35\times 10^{-13}$	0.0143	0.4809
[HHb]	$3.65\times 10^{-13}$	0.0395	-0.1021
[tHb]	$1.85\times 10^{-12}$	0.0160	0.3100

We then evaluated the functional efficacy of the predicted short channels using them as regressors in SCR to denoise long-channel signals. As shown in Table 9, the cleaned long-channel signals demonstrated strong agreement with those denoised using short-channel ground truth, particularly for $\left[O_{2}\mathrm{Hb}\right]$ (r=0.8478) and [tHb] (r=0.8844). These results indicate the successful preservation of task-related activity while suppressing noise.

**Table 9:** Long-channel SCR performance using predicted short-channel regressors in activation channels only. These metrics indicate model impact on task-related denoising.

Type	MSE	NMSE	r
$\left[O_{2}\mathrm{Hb}\right]$	$7.00\times 10^{-14}$	0.0140	0.8478
[HHb]	$2.50\times 10^{-14}$	0.0361	0.5810
[tHb]	$3.00\times 10^{-14}$	0.0109	0.8844

Finally, we analyzed residual variance in the long-channel signals following SCR in order to quantify the amount of remaining physiological noise. Table 10 compares the results across three configurations: SCR using ground-truth short channels; predicted short channels from preprocessed data (with rejection); and predicted short channels from raw data (without rejection). Predicted regressors from the rejected dataset yielded residual variances comparable to or lower than those obtained using ground-truth short channels, particularly for $\left[O_{2}\mathrm{Hb}\right]$ and [tHb].

**Table 10:** Residual variance in activation-related long channels after SCR. Variance is compared across regressors from predicted vs. ground-truth short channels, with and without channel rejection.

Type	Ground-truth Var	Predicted Var (with rejection)	Predicted Var (without rejection)
$\left[O_{2}\mathrm{Hb}\right]$	$1.481\times 10^{-13}$	$9.778\times 10^{-14}$	$1.447\times 10^{-13}$
[HHb]	$2.338\times 10^{-14}$	$2.788\times 10^{-14}$	$4.660\times 10^{-14}$
[tHb]	$8.764\times 10^{-14}$	$6.513\times 10^{-14}$	$1.099\times 10^{-13}$

**Fig. 5:**
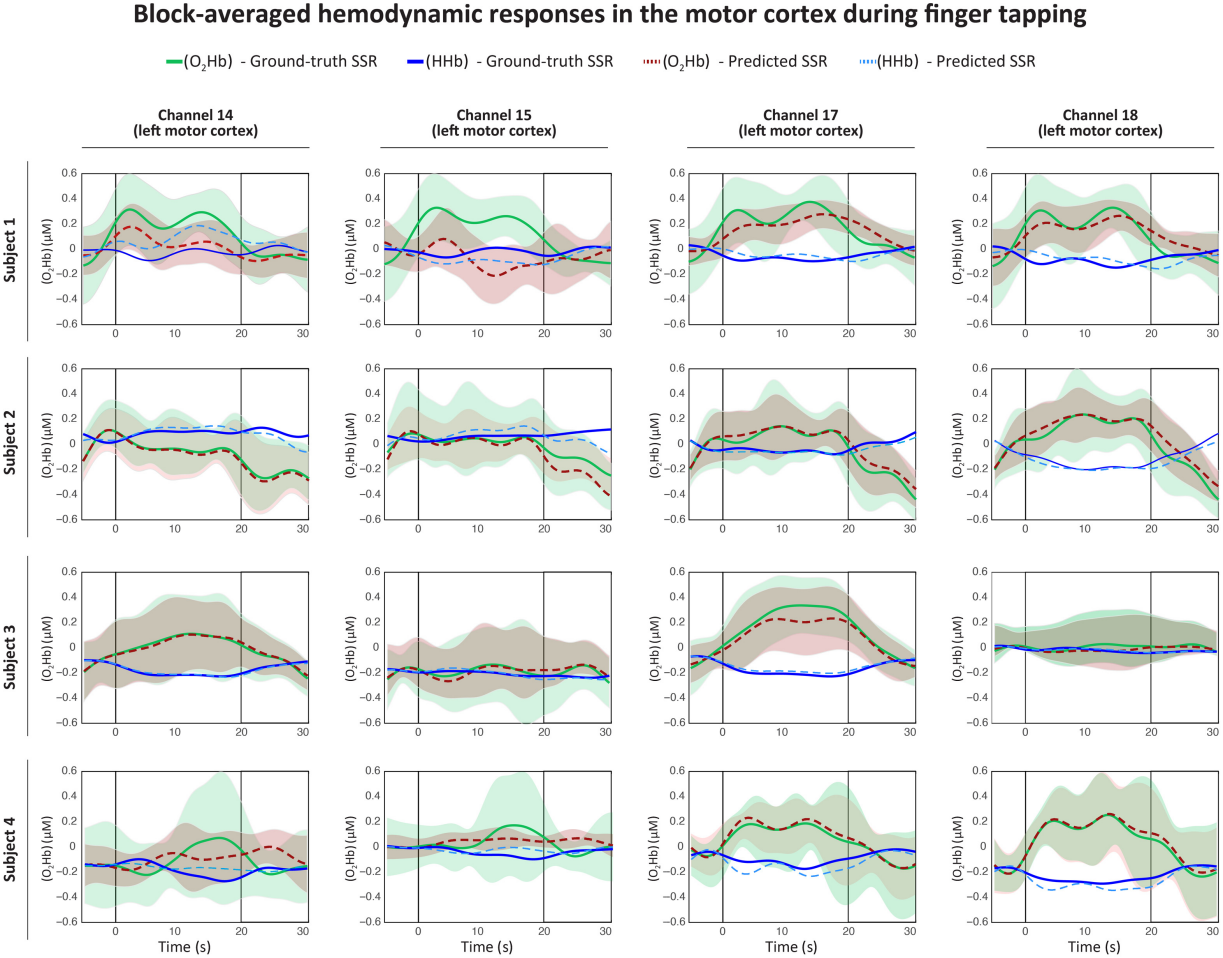
Block-averaged hemodynamic responses during the finger-tapping task for four motor cortex channels across four subjects. Each subplot displays the averaged time courses of $\left[O_{2}\mathrm{Hb}\right]$ and [HHb] concentrations recorded from motor-related long channels (channels 14, 15, 17, 18), calculated following short-channel regression using either ground-truth (solid lines) or predicted short-channel signals (dashed lines). Data are aligned to the onset of the motor task (0 s), with a 5-s baseline preceding the task (−5 to 0 s), followed by 20 s of finger tapping (0 to 20 s), and a 10-s post-task rest (20 to 30 s). Shaded regions around the $\left[O_{2}\mathrm{Hb}\right]$ curves indicate standard deviation across trials. Across all subjects and channels, both predicted and real short-channel regressors yield comparable hemodynamic patterns, indicating preserved task-related responses and effective systemic noise removal.

Figure 5 illustrates the task-related concentration dynamics across four motor cortex channels in four subjects. Each subplot shows the $\left[O_{2}\mathrm{Hb}\right]$ and [HHb] responses during finger-tapping blocks and compares the signals denoised using predicted and ground-truth short channels. The shaded areas denote the standard deviation across task blocks. The average response is summarised across multiple task blocks, spanning from 5 s pre-stimulus baseline to 30 s post-stimulus and including 20 s of active finger tapping.

## 4 Discussion

This study presents a novel deep learning approach to address a long-standing challenge in fNIRS: isolating and removing superficial physiological artifacts in the absence of physical short-separation channels. Consistently with recent reviews on transformer use in biosignal processing,^42^^,^^45^^–^^47^ we demonstrate the feasibility of predicting short-channel signals directly from long-separation fNIRS data using a transformer-based architecture. The model, which was trained on paired short-long channels recordings during the resting state, accurately reconstructs the extracerebral hemodynamic components typically captured by physical short-separation optodes.

In the following sections, we discuss the performance of the model across multiple test scenarios, including unseen resting-state datasets and a finger-tapping task. We also evaluated its effectiveness as a noise regressor in comparison to physical short channels and conventional preprocessing baselines.

### 4.1 Dataset 1

To assess the accuracy of short-channel prediction under resting-state conditions, we evaluated the similarity between the predicted and ground-truth short-channel OD signals using three quantitative metrics: MSE, NMSE, and Pearson correlation.^39^ The predicted short-channel traces demonstrated high correspondence with measured signals, with a median NMSE of 0.0471 and a Pearson correlation coefficient of r=0.70. These results indicate that the model effectively captures the superficial physiological components typically isolated by physical short-channels. In some cases, such as Fig. 3(a) (channel 1 at 850 nm), predicted and ground-truth short-channel signals diverged locally, likely due to noise or suboptimal coupling in the physical measurement.

Following OD-to-concentration conversion, predictive accuracy remained robust across chromophores. The median NMSE values ranged from 0.0263 to 0.0314, and Pearson correlations ranged from r=0.49 for [HHb] to r=0.67 for [tHb], indicating that the temporal dynamics of the predicted short-channel concentration signals closely match those of the ground truth. Although minor discrepancies are expected due to sensor noise and inter-channel anatomical variability, the predicted traces were sufficiently accurate for use in SCR pipelines, especially given their favorable signal-to-noise characteristics [Fig. 4(a)]. These observations support the use of transformer-predicted short channels as reliable surrogates for physical measurements in standard fNIRS preprocessing pipelines.

To further assess functional utility, we evaluated the impact of predicted short-channel regressors on long-channel SCR performance by computing the residual variance of long-channel signals after regression with either real or predicted short channels. Notably, predicted regressors yielded slightly lower residual variances across all three chromophores, with $\left[O_{2}\mathrm{Hb}\right]$ decreasing from $3.01\times 10^{-14}$ (ground truth) to $2.74\times 10^{-14}$ (predicted), and similar reductions were observed for [HHb] and [tHb]. This indicates that, at least in this dataset, the predicted regressors performed comparably to physical short channels, with marginal improvements that may reflect the ability of the model to generalize beyond measurement-specific noise. However, broader validation across larger and task-based datasets will be needed to confirm this observation.

### 4.2 Dataset 2

Dataset 2 served as a critical test of the ability of the model to generalize across different fNIRS acquisition systems and spatial configurations. This dataset introduced both a novel hardware setup and an expanded optode montage, increasing the number of long channels and introducing greater anatomical variability. These domain shifts posed a meaningful challenge to the model, making it an ideal setting to evaluate robustness to system-level differences.

Initial results confirmed that the transformer-based model could reconstruct short-channel OD signals with moderate fidelity even without preprocessing. As shown in Table 5, median performance without channel rejection yielded an NMSE of 0.1784 and a Pearson correlation coefficient of 0.2771. However, when wavelet coherence-based channel rejection was applied, performance improved substantially: NMSE decreased to 0.0875 and correlation increased to 0.4296. This finding reinforces the importance of rejecting unreliable channel pairs prior to model inference. Channels with poor coherence often introduce inconsistent noise patterns that compromise the ability of the transformer to learn stable temporal dependencies. Removing these inputs likely enhanced the capacity of the model to focus on physiologically plausible dynamics shared across the remaining clean long channels. Although prior work has employed wavelet transform coherence among long channels to detect systemic physiological artifacts, wavelet-based preprocessing has been recommended for motion and noise correction,^64^^–^^66^ and hyperscanning studies,^36^^,^^67^^–^^70^ our use of wavelet coherence between short- and long-separation channels for targeted channel rejection is a novel methodological contribution.

In the hemoglobin concentration domain, performance mirrored these trends. With preprocessing, the predicted short-channel signals closely followed ground-truth dynamics, especially for [tHb] (r=0.6099). This chromophore consistently yielded the highest performance across all datasets, likely due to its additive nature and reduced sensitivity to inversion errors during OD-to-concentration conversion. The relatively lower performance for [HHb] is consistent with its known vulnerability to low signal amplitude, greater variability, and interference from skin perfusion artifacts. Figure 4(b) shows that predicted short channels can be successfully used for SCR without distorting the shape of long-channel $\left[O_{2}\mathrm{Hb}\right]$ signals, demonstrating that SCR with virtual short channels closely replicates outcomes obtained with physical regressors.

Importantly, these findings confirm that the representations learned by the model of superficial hemodynamics are not specific to a particular hardware configuration or montage. Through fine-tuning of the input and output layers and brief exposure to a subset of new recordings, the transformer retained its internal attention structure while adapting to the expanded spatial layout of dataset 2. This suggests that most of the physiological patterns learned during training on dataset 1 are transferable, provided that initial layers are adjusted to accommodate new dimensional inputs. This capability is especially valuable in research settings that rely on legacy datasets lacking short-channel coverage, or in systems where short-separation detectors are sparse, inconsistently placed, or compromised by poor coupling.^16^^,^^66^ Its capacity to generate short-channel regressors even in datasets with limited or no physical short channels opens up possibilities for retrospective analysis.

These results collectively confirm that the transformer-based model retains its predictive capacity even when applied to fNIRS data collected on different hardware, provided that preprocessing steps such as channel rejection are applied. The results suggest that the model is not overfitted to the characteristics of a single system but rather learns transferable features of superficial hemodynamics that generalize across acquisition setups.

### 4.3 Dataset 3

Dataset 3 involved a block-designed finger-tapping task, characterized by spatially and temporally localized cortical responses.^71^ This evaluation provided an opportunity to test the robustness of the model in a dynamic, non-resting paradigm. The ability of the model to predict short-channel signals during movement-based tasks, where physiological artifacts are more pronounced, demonstrates its generalization beyond resting-state conditions. Unlike resting-state data, motor paradigms generate well-defined activation patterns, enabling a more targeted assessment of the utility of the model in supporting SCR without compromising neural signals of interest.

As in the resting datasets, preprocessing played a pivotal role. Specifically, channel rejection based on wavelet coherence proved essential to ensuring signal fidelity. When low-quality long–short-channel pairs were not excluded, the performance of the OD prediction declined markedly (Pearson r dropped from 0.4061 to 0.0764), replicating the same pattern observed in dataset 2. This reinforces the idea that coherence filtering acts as a critical epicenter for model reliability, ensuring that the training and inference data are grounded in physiologically meaningful signal relationships.

Predictions for chromophore concentrations showed acceptable performance for $\left[O_{2}\mathrm{Hb}\right]$ and [tHb], while [HHb] exhibited notably lower correlation and higher variance. This finding is consistent with prior reports that [HHb] signals tend to have lower signal-to-noise ratios and are more susceptible to superficial artifacts and spectral degradation. Interestingly, block-averaged results showed that task-aligned changes in $\left[O_{2}\mathrm{Hb}\right]$ remained preserved even when predicted short channels were used for SCR, indicating that the model-generated regressors effectively suppressed physiological noise without compromising the task-evoked response.

The effectiveness of predicted short channels in SCR was confirmed quantitatively: denoised $\left[O_{2}\mathrm{Hb}\right]$ and [tHb] long-channel signals showed high temporal correlations (r=0.8478 and r=0.8844, respectively), comparable or superior to SCR performed with ground-truth short channels. Notably, residual variance analysis revealed that SCR using predicted regressors derived from the channel-rejected dataset yielded the lowest or near-lowest residuals, including for [HHb]. This finding highlights the benefit of combining robust preprocessing with data-driven modeling, where predictive stability often outperforms noisy measurements.

Moreover, Fig. 5 provides additional insights into model performance across motor cortex channels and subjects. In many channels, the predicted SCR traces show reduced trial-by-trial variability, as indicated by the narrower standard deviation bands compared to those using ground-truth short channels. This should not be interpreted as evidence that predicted short channels are superior, but rather that the model produces regularized regressors less affected by measurement-specific noise. The effect is unlikely to reflect overfitting to the long channels, given the use of dropout, data augmentation, and strict train/test separation, but further validation will be required to determine whether such regularization is advantageous across broader datasets. This result suggests that predicted regressors may provide a more regularized approximation of superficial signal components, yielding cleaner estimates of task-related cortical responses. In some cases, however, larger discrepancies were observed (e.g., subject 1/channel 15 and subject 4/channel 15), likely reflecting inter-subject variability and, in the latter case, high variance in the ground-truth SSR. These localized differences suggest that while predicted regressors are smoother and less affected by noise, they may diverge from unstable short-channel signals. As shown in Fig. 5, both SCR approaches, using real and predicted short channels, yield highly comparable task-evoked profiles, characterized by the expected increase in $\left[O_{2}\mathrm{Hb}\right]$ and decrease in [HHb] during motor execution in many channels. Importantly, the predicted regressors preserve the temporal dynamics and amplitude of the canonical hemodynamic response, with minimal distortions or delays.

These findings support broader adoption of virtual SCR in fNIRS pipelines in task-based paradigms, providing robust denoising even under increased physiological variability, particularly in legacy datasets or systems lacking dense short-separation coverage.^72^

### 4.4 Parallels in fMRI Physiological Noise Correction

The use of ML to generate surrogate regressors for physiological noise correction has also gained traction in fMRI research. In a 2020 study, researchers trained a deep convolutional generative adversarial network (DCGAN) to fill in BOLD signal loss (e.g., caused by metal implants or missing data) and found that the reconstructed time-series and functional connectivity patterns closely matched the original data.^73^ Recent work in BOLD signal processing has shown that deep learning architectures, such as CNNs, LSTMs, and transformers have been successfully trained to predict cardiac and respiratory waveforms directly from fMRI time series, allowing for retrospective physiological noise correction even in the absence of external recordings.

For instance, Aslan et al. developed a CNN-based method to extract the cardiac pulsation waveform from simultaneous multi-slice fMRI data.^74^ Their approach produced high-fidelity cardiac signals from the BOLD data itself, which are used to regress out pulsatile noise in the absence of a pulse oximeter.

Similarly, several groups have trained deep models to recover respiratory fluctuations from fMRI. Salas et al. and Bayrak et al. utilized CNN/U-Net and LSTM architectures to infer respiratory volume variation (RV) signals from resting-state fMRI, showing that slow breathing-related oscillations are captured in the imaging data.^75^^–^^77^ Although those initial models struggled to capture the extremes of the breathing waveform (e.g., signal segments at scan start/end), subsequent improvements have closed this gap.

Notably, Addeh et al. introduced a model employing three separate CNNs to reconstruct continuous respiratory waveforms across entire fMRI scans.^78^ By training on a large pediatric dataset with many motion artifacts, their method achieved robust RV signal predictions even in challenging conditions. Adding head-motion estimates as additional inputs further improved the fidelity of the predicted respiratory signals, highlighting that motion parameters contain useful information about breathing-induced variations.

These approaches align closely with our method, where a transformer model predicts short-channel regressors from long-channel fNIRS signals, effectively learning to isolate superficial physiological components. Such convergence across imaging modalities highlights a growing confidence in data-driven surrogate generation as a robust alternative to direct physiological measurement, an encouraging sign for wider adoption of virtual short-channel regression in fNIRS.

### 4.5 Choice and Impact of Fixed Numerical Parameters

The predictive performance of any deep learning model depends not only on the overall architecture but also on the choice of fixed numerical parameters throughout the training and inference pipeline. In our case, several hyperparameters, ranging from preprocessing thresholds to model configuration settings, were empirically chosen based on prior domain knowledge, existing literature, or grid search optimization. Below, we provide the rationale for each parameter and speculate on the expected effects of varying them.

#### 4.5.1 Window length

Our transformer model selected for fNIRS signal reconstruction uses an embedding dimension of 128, four attention heads, and two encoder layers. This configuration was chosen based on an extensive grid search across 12 architectures (see Table S5 in the Supplementary Material). It offers a balance between signal fidelity and computational efficiency, achieving near-peak performance while remaining lightweight enough for training and inference on resource-constrained hardware such as Google Colab’s T4 GPUs. Although larger models (e.g., embed_dim = 256) showed slight performance improvements, they incurred significantly higher memory demands, slower inference times, and smaller batch sizes. Notably, the 128-dimensional embedding delivers 94% of the performance of the 256-dimensional model while requiring only 30% of the parameters. We chose 128 samples (∼18.2  s at 7 Hz) as the temporal window for input segmentation based on a grid search comparing different lengths. This duration captures both slow systemic fluctuations (e.g., Mayer waves) and typical task-evoked fNIRS responses. Shorter windows (e.g., 64 samples) led to underfitting due to reduced temporal context. Bang et al. similarly extracted features from 128-sample EEG epochs (1 s at 128 Hz).^79^ Such window sizes capture transient responses while maintaining manageable input length.

In fNIRS tasks, window lengths around 10 to 20 s (which at ∼7 to 10 Hz sampling yield ∼70 to 200 samples) are often used to capture full hemodynamic responses. Eastmond et al. reviewed that increasing window length from 1 s to 10 s improved classification accuracy (from ∼70% to ∼77%), and 15 to 30 s windows yielded >85% accuracy in some fNIRS BCI tasks.^80^ This suggests that a window of 128 samples (e.g., ∼12 to 18 s in fNIRS) is reasonable for capturing hemodynamic patterns while not averaging out temporal dynamics. Shorter windows offer finer temporal resolution but risk missing slow trends, whereas longer windows improve SNR at the cost of fewer training segments. Liu et al. found classification of mental workload peaked around a 10-s window.^81^ Thus, a 128-sample window (on the order of 10 to 20 s in typical fNIRS sampling) aligns with common practice for balancing context length and stationarity of the signal.

#### 4.5.2 Feedforward layer size

The size of the feedforward sublayer in each encoder block was set to 256, representing a 2× expansion over the embedding dimension of 128. This follows the standard design principle in the original transformer model, which recommends 2 to 4× expansions^50^ and was supported by a grid search (Table S5 in the Supplementary Material). The training dataset comprised 1311 samples in total (1048 for training, 131 for validation, and 132 for testing), providing sufficient examples for stable optimization. To control for the potential risk of overfitting given the relatively small size typical of fNIRS datasets, we applied dropout (10%), weight decay ($10^{-4}$), and test-time augmentation. Training and validation losses remained closely aligned, and performance generalized well to two independent external datasets, indicating that the model did not overfit. Larger expansions (e.g., 512) produced only marginal improvements, but considerably increased parameter count and computational cost. The 128/256 configuration therefore provided near-optimal accuracy while remaining efficient and robust. Although similar settings have been reported in EEG transformers (e.g., Medformer,^82^ GET^83^), our choice was primarily based on empirical validation within the present fNIRS datasets.

#### 4.5.3 Dropout rate

A dropout rate of 10% was adopted as a regularization strategy during training.^84^ This value is supported by prior neuroimaging applications of deep networks, where it has been shown to improve generalization in small-to-medium datasets (e.g., fNIRS and EEG).^83^^,^^85^ For example, Hu et al. applied a 10% dropout after LSTM layers in an fNIRS classifier. Yu et al. found that using dropout 0.1 in an EEG emotion model improved generalization.^86^ Likewise, a cross-subject EEG graph network used dropout 0.1 to randomly disable neurons during training.^87^ These reports indicate that a dropout rate of 0.1 offers an optimal balance; higher rates (e.g., 0.5) impair learning on small datasets, whereas 0.1 provides effective regularization with minimal impact on convergence.^86^

#### 4.5.4 Learning rate

The initial learning rate for the AdamW optimizer was selected through focused grid search over learning rate $\in \{1\times 10^{-3},8\times 10^{-4},5\times 10^{-4},1\times 10^{-4}\}$ and weight decay $\in \{1\times 10^{-3},1\times 10^{-4},1\times 10^{-5}\}$, with the best combination found to be $\text{learning rate}=8\times 10^{-4}$ and $\text{weight}\;\text{decay}=1\times 10^{-4}$. The AdamW optimizer has become popular in biomedical deep learning due to its improved generalization performance.^88^ By decoupling weight decay from the gradient updates, AdamW avoids the overshoot and generalization issues sometimes seen with standard Adam.^89^ Researchers often choose a relatively small initial learning rate for AdamW. In fact, an initial learning rate of 0.0008 has been empirically found to yield fast convergence without destabilization in many domains.^90^ In conjunction with AdamW, many studies employ cosine annealing learning rate schedules to further improve convergence and generalization. Cosine annealing gradually reduces the learning rate following a cosine curve (often over each epoch or training cycle) rather than dropping it abruptly.

#### 4.5.5 Data augmentation parameters

Data augmentation strategies were tailored to fNIRS characteristics, incorporating realistic noise levels, temporal shifts, amplitude scaling, and baseline perturbations to improve robustness across both training and test-time conditions, following established practices for deep learning data augmentation.^91^

Our test-time augmentation strategy incorporated Gaussian noise (σ=0.01), temporal jittering (±10 samples), amplitude scaling (±5%), and baseline shifts. These values were chosen to simulate realistic physiological variations and match previously published augmentation protocols for EEG and fNIRS signals.^59^^,^^92^ Adding slight Gaussian noise to a time-series is a well-known augmentation to improve generalization.^93^ With signals normalized to [−1,1], a 0.01 standard deviation adds only 1% noise. Zhao and Fan used this as a mild EEG augmentation.^94^ Empirical surveys confirm that noise levels on the order of 1% to 5% of the signal range effectively augment data without overwhelming the true signal.^95^ However, if σ is too large (>0.2), performance degrades as the physiological signal gets drowned out. Thus, σ=0.01 is a conservative choice widely used for data augmentation.

Time-shifting or slight misalignment of signals is another augmentation that preserves sequence content but tests model temporal tolerance. A ±10-sample jitter at 7 Hz (≈±1.4  s shift) is relatively mild. Um et al. introduced random time shifts in wearable sensor data to augment training, though they caution that large shifts distort temporal dependencies.^59^

Scaling the signal by a random factor is a common augmentation to simulate inter-subject differences. Zhao and Fan used random multipliers 0.9 to 1.1 (±10%) for EEG data, and an extensive survey by Iwana and Uchida confirms scaling by a factor sampled from a narrow Gaussian (mean 1, σ around 0.1 or less) is effective.^93^^,^^94^ Our choice of ±5% is even more subtle, ensuring the shape of the hemodynamic curve is preserved while introducing slight amplitude variation.

#### 4.5.6 Bandpass filter range

A bandpass of ∼0.01 to 0.2 Hz is very commonly applied in fNIRS to isolate the neural hemodynamic frequencies. The lower cutoff (∼0.01  Hz) removes baseline drifts and very slow oscillations, whereas the upper cutoff (∼0.2  Hz) removes respiration (∼0.2 to 0.4 Hz) and cardiac pulsations (∼1  Hz). Hu et al. explicitly used a 0.01 to 0.2 Hz bandpass to remove physiological noises.^63^ Other recent fNIRS studies employ a similar filter. For instance, Khan et al. used a 0.01 to 0.2 Hz bandpass as part of preprocessing.^96^ Pinti et al. noted that a Butterworth bandpass filter at 0.01 to 0.2 Hz is standard practice and effectively separates neural signals from systemic noise.^97^

#### 4.5.7 Downsampling

The sampling rate was resampled uniformly to 7 Hz to match the acquisition frequencies of the included datasets (NIRSport 1 and 2). This downsampling step simplifies modeling and reduces computational cost while preserving the information content relevant to typical fNIRS analyses (<1  Hz). Sampling below 4 Hz may risk aliasing faster physiological rhythms, whereas higher rates increase data size without practical benefits. It is common to downsample fNIRS data to somewhere in the 4 to 10 Hz range after acquisition. Many fNIRS devices natively sample at ∼5 to 10 Hz; e.g., some systems collect at ∼5  Hz by default.^6^ Our choice of 7 Hz lies in this typical range. It retains the important oscillations (heartbeat is ∼1  Hz, respiratory ∼0.2 to 0.3 Hz, Mayer waves ∼0.1  Hz) while greatly reducing data size and high-frequency noise.

#### 4.5.8 Wavelet coherence threshold

To enhance the robustness of input data against physiological and hardware-related noise, we implemented a wavelet coherence–based filtering strategy to identify and reject long fNIRS channels that consistently exhibited low temporal coherence with their corresponding short-distance channels. The underlying assumption is that long channels lacking strong coherence with proximal short channels are likely contaminated by artifacts, particularly in resting-state recordings. To determine an appropriate coherence threshold for rejecting noisy long channels, we conducted a thorough evaluation across multiple threshold values: {0.20, 0.30, 0.39, 0.45, 0.50}. For each threshold, the model was retrained from scratch and evaluated on a held-out set of participants using mean absolute error (MAE) and Pearson correlation between predicted and target signals. Our results show that very low thresholds (e.g., 0.20) retain nearly all channels (mean rejection: ∼0.2 channels/subject), but yield poor predictive performance (MAE: 0.0133; Pearson r: 0.5670), likely due to the inclusion of noise-contaminated channels. By contrast, higher thresholds (e.g., 0.45 to 0.50) lead to more aggressive rejection (up to ∼4 channels/subject) and modest MAE improvements (e.g., 0.0084 at 0.50), but at the cost of reduced signal quality, as reflected by plateauing or declining Pearson correlation scores (e.g., 0.5787 at 0.50 vs. 0.5896 at 0.39). A coherence threshold of 0.39 emerged as the most effective compromise. It achieved the highest Pearson correlation (r=0.5896) across all tested thresholds, alongside a favorable MAE (0.0113) and moderate channel rejection (∼2 channels/subject). This suggests that a threshold of 0.39 effectively suppresses non-informative or noisy signals while preserving meaningful hemodynamic patterns essential for accurate prediction. This empirical choice is further supported by subject-level analyses: in several cases, correlation with ground truth improved markedly at the 0.39 threshold compared with both more lenient and more aggressive filtering. Notably, although stricter thresholds (e.g., 0.50) may reduce MAE further, they do not enhance correlation with the ground truth, indicating potential loss of physiologically relevant signal content. Therefore, we selected 0.39 as the optimal coherence threshold, balancing predictive accuracy, signal integrity, and data retention. Detailed performance metrics (mean MAE, mean Pearson r, and mean $R^{2}$ across all test subjects of testing dataset 1) are reported for each threshold configuration in Table S4 in the Supplementary Material.

#### 4.5.9 Impact of motion artifact correction

We explored the effect of applying wavelet-based motion artefact (MA) correction to long-channel signals prior to model inference using a standard threshold of 0.1. Surprisingly, the results indicated that MA correction had a minimal impact on prediction accuracy. When tested on dataset 1, the model achieved almost identical performance with and without this preprocessing, with a mean MAE of 0.0112 (vs. 0.0113) and a mean Pearson correlation of 0.5760 (vs. 0.5896). The predicted short channels yielded comparable, if not better, metrics when MA correction was omitted from the preprocessing pipeline. The most notable difference emerged in SCR performance: Pearson correlation values for the regressed-out long channels were greater than 0.70 for all chromophores without MA correction, compared with substantially lower values when correction was applied ($\left[O_{2}\mathrm{Hb}\right]$: 0.4581, [HHb]: 0.5945, [tHb]: 0.4717). The full results are reported in Tables S1–S3 in the Supplementary Material. These findings suggest that the transformer may implicitly learn to filter out motion-related noise during training, thus reducing the need for explicit artefact correction in this context.

### 4.6 Evaluation Strategy and Choice of Architecture

The evaluation procedure adopted in this study was designed to ensure both reproducibility and computational feasibility while maintaining sufficient statistical power for model optimization. Rather than employing conventional cross-validation procedures (e.g., k-fold or subject-wise), the dataset was partitioned into training, validation, and test sets using a fixed 80/10/10% split, which allowed stable optimization and consistent benchmarking across experiments. Given the relatively small sample size typical of fNIRS datasets and the computational demands of Transformer architectures, this strategy provided a practical balance between robustness and efficiency. Moreover, model generalizability was examined using two independent external datasets collected with different systems and experimental paradigms, offering a complementary validation beyond the primary dataset.

The choice of a Transformer-based model over more conventional architectures such as CNNs or U-Nets was guided by its ability to jointly model temporal and spatial dependencies across multiple channels. CNNs and U-Nets are effective in capturing local spatial patterns, yet their limited receptive fields make them less suitable for learning the broader temporal context intrinsic to fNIRS signals unless specifically engineered with deep or dilated layers. By contrast, the self-attention mechanism of Transformers enables global integration of temporal and spatial features, facilitating the representation of multi-scale systemic fluctuations that characterize short-channel signals. This architectural flexibility, combined with parallel processing efficiency, makes the Transformer particularly well suited for virtual short-channel generation and related fNIRS preprocessing tasks.

### 4.7 Limitations

Although the proposed transformer-based model offers promising capabilities for predicting short-channel signals from long-separation fNIRS data, several limitations must be acknowledged to guide future improvements and applications.

First, the model is intrinsically dependent on the quality of the input long-channel signals. Because the prediction pipeline is entirely data-driven, any residual motion artifacts, physiological noise, or signal degradation within the long channels directly impair the accuracy of the reconstructed short-channel outputs. This constraint highlights the critical importance of rigorous preprocessing both during model training and during deployment at inference time, a concern echoed in previous studies emphasizing the value of wavelet-based and systemic-noise-aware preprocessing pipelines.^65^^,^^66^

Second, the current model architecture assumes a fixed montage configuration: that is, it learns spatial and temporal patterns based on the specific arrangement of sources and detectors in the training dataset. Consequently, its generalizability is demonstrated only within the scope of the datasets tested here, specifically across different NIRSport systems and between resting-state and task-based paradigms. Broader generalization to alternative probe layouts, clinical populations, or recording contexts will require further validation. Although fine-tuning on a subset of new data partially mitigates this issue, this process requires the presence of physical short channels in the new configuration, which undermines the goal of fully hardware-independent preprocessing. This limitation points to the need for architectural modifications, specifically, the integration of spatial attention mechanisms or graph-based encodings that allow the model to represent inter-channel relationships in a layout-agnostic manner. Similar strategies have been proposed in EEG and biosignal literature to enhance the spatial adaptability of deep learning models.^42^^,^^46^^,^^47^ Such improvements would enable the model to adapt to variable probe geometries without retraining from scratch or relying on auxiliary short-channel data. Similarly, domain adaptation techniques or training on multi-layout datasets would enhance robustness across diverse experimental settings.

Third, in its current form, the model requires access to at least a subset of data recorded with both long- and short channels for fine-tuning before it can be reliably applied to new montages or systems. This dependency currently limits immediate out-of-the-box deployment. In the long term, the creation of large-scale, multi-site datasets with full-head short-channel coverage will be essential to enable the development of community-wide pretrained models that can be broadly adopted without additional calibration.

Fourth, the model currently predicts short-channel signals only from existing long channels and does not incorporate auxiliary physiological signals (e.g., cardiac, respiratory, or blood pressure traces) that could further improve disambiguation of neural versus systemic contributions. Although our model focuses exclusively on optical input, future extensions could integrate multimodal data streams or physiological priors into a unified regression framework to further boost denoising performance in settings with high systemic interference. Prior work has shown that systemic confounds may distort fNIRS-derived neural inferences and may not always be removable via optical channels alone.^13^^,^^14^^,^^72^

Fifth, to architectural and data-related considerations, another limitation concerns the use of fixed numerical parameters throughout the model and preprocessing pipeline. Although the fixed numerical parameters used in this study were chosen based on empirical tuning, grid search, and domain-informed heuristics, they have not yet been systematically optimized through formal sensitivity analyses. This presents a limitation to generalizability. Small changes in parameters such as the temporal window, dropout, or filter boundaries may affect the ability of the model to capture slower or faster physiological dynamics or influence the trade-off between bias and variance. Similarly, although our wavelet coherence threshold was empirically selected based on predictive performance, different thresholds might yield better results under alternative noise conditions or hardware configurations. Data augmentation parameters were designed conservatively, but overly strong augmentations may distort temporal features in future applications. Without extensive cross-validation across parameter configurations, it is difficult to determine the robustness of the current model beyond the settings explored here. Although we adopted a consistent preprocessing pipeline across datasets, different artifact correction methods or parameter settings, such as alternative MA removal thresholds or filtering strategies, may yield different results. Therefore, future work should include sensitivity analyses to assess the stability of predictions concerning parameter changes and potentially incorporate hyperparameter optimization routines or adaptive learning schemes. This will be essential to ensure the generalizability and reproducibility of the proposed framework across broader neuroimaging contexts.

Sixth, the lower correlations observed in the task-based dataset (Table 8) should be interpreted with caution, as they reflect the very small sample size (N=4) and the greater susceptibility of task-related recordings to motion artifacts and low-SNR hemoglobin signals. Although the model was still able to preserve task-evoked responses (Table 9, Fig. 5), larger task-based datasets will be required to more comprehensively validate performance under active conditions.

In addition, we did not include a direct comparison with computationally efficient and widely established approaches such as PCA or global signal averaging, which are often used to approximate superficial regressors in the absence of physical short channels. Prior studies have shown that while PCA and global averages capture global components, they cannot reproduce the local heterogeneity of scalp physiology as effectively as short channels.^25^^,^^31^^,^^34^^,^^97^ Because our model was validated against measured short channels and shown to approximate them closely, this provides indirect evidence that it is likely to outperform PCA and global averages, which are known to be less effective than SC regression. Future work will include a systematic benchmarking to directly quantify this added value, ideally using datasets with full-head coverage and dense SC placement to provide an optimal reference. Although deep learning is initially more complex, once trained, such models can be readily integrated into existing fNIRS toolboxes (e.g., Homer, Cedalion), facilitating adoption by the broader community.

Finally, the model does not currently estimate uncertainty in its predictions. In practical applications, especially in clinical or real-time settings, it would be valuable to accompany each predicted short-channel signal with a measure of confidence or variance. Bayesian transformer architectures or ensemble modeling could provide calibrated uncertainty estimates, offering users a principled way to assess the trustworthiness of virtual short-channel regressors on a per-channel basis.

## 5 Conclusion

This study presents a transformer-based deep learning model capable of predicting short-channel OD fNIRS signals directly from long-channel data, offering a practical and generalizable solution for systemic noise correction in both resting-state and task-based paradigms. Across three independent datasets, including cross-system recordings and motor tasks, the model demonstrated strong agreement with ground-truth short-channel signals.

The self-attention mechanism of the transformer proved particularly effective in capturing the spatial and temporal dependencies inherent in fNIRS time series, outperforming recurrent architectures known to struggle with long-range dependencies. Predicted short-channel signals maintained fidelity across both OD and hemoglobin concentration domains, preserved task-evoked hemodynamic responses, and consistently reduced residual physiological variance in long-channel signals.

Although these outputs are model-derived rather than empirically acquired, they represent a reliable and interpretable approximation of superficial physiological components. When applied with transparency and within clearly defined limitations, they offer a viable alternative for SCR in scenarios where physical short-separation measurements are unavailable or impractical.

Importantly, this virtual regression framework addresses several limitations of physical short channels, including hardware constraints, non-ideal optode placement, and limited scalp coverage.

Our findings suggest that, when supported by preprocessing such as channel rejection, transformer-based predictions serve as robust stand-ins for measured short-channel signals, enabling both forward-looking applications and retrospective correction of previously collected datasets.

Future work will broaden validation efforts across diverse populations and hardware configurations, including cases where short channels are located farther from long-channel sources, a common challenge in sparse or nonstandard probe layouts. The framework will also be extended to incorporate alternative deep learning architectures (e.g., LSTMs, CNNs) and multimodal fusion with auxiliary physiological signals, with the goal of improving generalization, interpretability, and integration into standardized fNIRS preprocessing pipelines. In addition, a key direction for future work will be to extend the model toward full denoising of long-channel signals, enabling an integrated, end-to-end preprocessing solution.

## Supplementary Material

10.1117/1.NPh.12.4.045008.s01

## Data Availability

The data are not publicly available due to legal and ethical reasons. The code associated with this manuscript is available at https://github.com/vbankers/fNIRSTransformer
