# Supplementary material for: Transformer-based deep learning model for predicting fNIRS short-channel signals
Source: Neurophotonics. 2025 Nov 14;12(4):045008. doi: 10.1117/1.NPh.12.4.045008 (PMC12618017; doi:10.1117/1.NPh.12.4.045008)
Supplement: Supplementary file 1 [file NPh_012_045008_SD001.pdf]

Supplementary to: A transformer-based deep learning model for predicting fNIRS short-channel signals

Sabino Guglielmini,[a,*,#], Vittoria Banchieri,[a,#] Felix Scholkmann,[a,b,c] Martin Wolf[a,c]
[a] Biomedical Optics Research Laboratory, Department of Neonatology, University Hospital Zurich, University of Zurich, Zurich, Switzerland
[b] Institute of Complementary and Integrative Medicine, University of Bern, Bern, Switzerland
[c] Neuroscience Center Zurich, University of Zurich and ETH Zurich, Zurich, Switzerland

# Shared first authorship; * Sabino Guglielmini, E-mail: Sabino.Guglielmini@usz.ch

Motion artifact correction

To evaluate the effect of motion artifact (MA) correction on short-channel prediction, we applied a wavelet-based motion correction algorithm using the standard Homer2 software function, *hmrMotionCorrectWavelet* to the long-channel signals prior to model training and inference. The aim was to determine whether explicit MA correction improves the quality of the predicted short-channel signals.

Table S1: Performance of predicted versus actual short-channel OD signals.
Median values of mean squared error (MSE), normalized mean squared error (NMSE), and Pearson correlation coefficient (*r*) across all test subjects are reported. This table corresponds to results obtained when motion artifact correction was applied.

Metric	MSE	NMSE	r
OD	0.0001	0.0355	0.5980

Table S2: Performance comparison of long-channel signals following short-channel regression (SSR).
This table compares SSR outcomes using predicted versus ground-truth short-channel regressors, focusing on denoising efficacy. This table corresponds to results obtained when motion artifact correction was applied.

Type	MSE	NMSE	r
[O_2Hb]	1.0×10^{-14}	0.0199	0.4581
[HHb]	1.0×10^{-15}	0.0194	0.5945
[tHb]	1.0×10^{-14}	0.0242	0.4717

Table S3: Performance of predicted versus actual short-channel concentrations.
This table reports signal fidelity across [O_2Hb], [HHb], and [tHb] chromophores, computed after OD-to-concentration conversion using the modified Beer–Lambert law. Metrics are calculated using the predictions.

Type	MSE	NMSE	r
[O_2Hb]	7.0×10^{-14}	0.0389	0.3875
[HHb]	2.0×10^{-14}	0.0300	0.2788
[tHb]	1.3×10^{-13}	0.0305	0.4060

These findings, compared with the result of testing dataset 1 (Section 3.1), suggest that the transformer model effectively internalizes a denoising function during training, learning to extract and reconstruct the short-channel component from the long-channel signal even in the presence of substantial motion artifacts.

Wavelet coherence threshold

To determine the optimal threshold for wavelet coherence-based channel rejection, we conducted a systematic evaluation of five candidate thresholds: 0.20, 0.30, 0.39, 0.45, and 0.50. For each value, the training and evaluation process was repeated, and the prediction performance was assessed using mean absolute error (MAE), Pearson correlation coefficient (*r*), and coefficient of determination (R^2).

Table S4 reports the averaged results across subjects for each threshold. Low thresholds (e.g., 0.20) retained nearly all channel pairs but performed poorly, with decreased correlation and increased error, likely due to the inclusion of noisy or mismatched channels. Conversely, high thresholds (e.g., 0.50) rejected many channels and led to only modest MAE improvements but resulted in a decline or plateau in correlation with ground-truth signals.

A threshold of **0.39** emerged as the optimal balance, offering the best correlation (r = 0.5896) and competitive MAE (0.0113), while discarding only ~2 channels per subject. This suggests that 0.39 maximizes signal quality without sacrificing too much data. Consequently, this value was adopted throughout the main analysis and is considered empirically validated.

Table S4: Model performance across different wavelet coherence thresholds.
We evaluated five thresholds (0.20 to 0.50) for filtering low-quality channels based on their coherence with proximal short channels. The selected threshold of 0.39 is highlighted as it provides the best trade-off between accuracy and channel retention.

Threshold	Mean MAE	Mean R^2	Mean r
0.20	0.0133	-3.6896	0.5670
0.30	0.0118	-3.5622	0.5776
**0.39**	**0.0113**	**-3.8303**	**0.5896**
0.45	0.0098	-3.5013	0.5823
0.50	0.0084	-2.8173	0.5787

Model architecture selection and performance

To identify the optimal transformer configuration for predicting short-channel fNIRS signals, we performed a structured grid search across 12 architectural variants. The search varied three key hyperparameters: embedding dimension (64, 128, 256), number of attention heads (2 or 4), and number of encoder layers (1 or 2).

Table S5 details the performance of each configuration, reporting R^2 score, mean absolute error (MAE), Pearson correlation coefficient (*r*), and total parameter count. Among the tested configurations, dim = 128, heads = 4, layers = 2 was selected for final deployment, achieving a correlation of 0.8910 with a manageable parameter count (~262K). While the most complex model (dim = 256, heads = 4, layers = 2) reached slightly higher scores (r = 0.9127), it required more than triple the parameters and considerably more memory.

The selected model delivered >94% of the best performance at a fraction of the computational cost, making it ideal for training on limited hardware (e.g., Google Colab T4 GPUs). This trade-off between performance and efficiency was key in ensuring that the model remains practical for widespread use and potential real-time applications.

Table S5: Grid search results for the transformer architecture configurations.

This table summarizes the results of a grid search over model depth, embedding dimension, and attention heads. The chosen configuration (dim=128, heads=4, layers=2) is shown in bold.

Configuration	R^2 Score	MAE	Pearson *r*	Parameter Count
dim=64, heads=2, layers=1	0.6214	0.3845	0.8111	~49K
dim=64, heads=2, layers=2	0.6834	0.3535	0.8436	~81K
dim=64, heads=4, layers=1	0.6444	0.3772	0.8188	~53K
dim=64, heads=4, layers=2	0.7034	0.3453	0.8517	~89K
dim=128, heads=2, layers=1	0.7369	0.3259	0.8654	~131K
dim=128, heads=2, layers=2	0.7818	0.2951	0.8894	~230K
dim=128, heads=4, layers=1	0.7424	0.3216	0.8683	~147K
**dim=128, heads=4, layers=2**	**0.7855**	**0.2920**	**0.8910**	**~262K**
dim=256, heads=2, layers=1	0.8034	0.2807	0.8987	~459K
dim=256, heads=2, layers=2	0.8368	0.2500	0.9163	~787K
dim=256, heads=4, layers=1	0.8056	0.2742	0.8994	~523K
dim=256, heads=4, layers=2	0.8305	0.2565	0.9127	~915K
